# Incidental observation of bone modification by *Crematogaster* cf. *liengmei* (Hymenoptera: Formicidae) in Cape Town, South Africa

**DOI:** 10.1007/s12024-023-00714-2

**Published:** 2023-09-13

**Authors:** Adeyemi Daniel Adetimehin, Calvin Gerald Mole, Devin Alexander Finaughty, Marise Heyns

**Affiliations:** 1 Division of Forensic Medicine and Toxicology, Department of Pathology, University of Cape Town, Cape Town, South Africa; 2 Division of Natural Sciences, School of Chemistry and Forensic Science, University of Kent, Canterbury, UK; 3 Division of Clinical Anatomy and Biological Anthropology, Department of Human Biology, University of Cape Town, Cape Town, South Africa; 4 School of Medicine, Faculty of Life and Health Sciences, Ulster University, Derry/Londonderry, UK

**Keywords:** Ants, Forensic entomology, *Crematogaster* cf. *liengmei*, Insect scavenging, Taphonomy

## Abstract

Different taxa of insects have been reported to modify the skeletal remains of vertebrates during feeding or the creation of their larval and pupal chambers. Anecdotal reports on the modification of skeletal remains by ants exist but are rare. In this paper, a case of modification of bone due to the feeding activity of the ant, *Crematogaster* cf. *liengmei* (Hymenoptera: Formicidae), is reported. In November 2022, a bone was encountered incidentally in the vicinity of the Table Mountain National Park. The bone had been colonized by several individuals of *Crematogaster* cf. *liengmei*. Several ants were observed feeding on and removing bone particles and soft tissue remnants. A closer observation revealed striae/furrows on the epiphyseal ends of the bone. A 60-kg pig cadaver used in a winter decomposition trial in the same area was similarly colonized by this ant species, resulting in soft-tissue modification. Due to the similarity in the striae/furrows observed on the bone and the bitemarks inflicted by the ants on the right ear of the pig cadaver in the vicinity, it was concluded that the striae/furrows seen on the bone were created by the ants. Our observations show that *Crematogaster* cf. *liengmei* is an important taphonomic bio-agent that can contribute to the modification of skeletal remains in terrestrial environments.

**Supplementary Information:**

The online version contains supplementary material available at 10.1007/s12024-023-00714-2.

## Case report

**Fig. 1:**
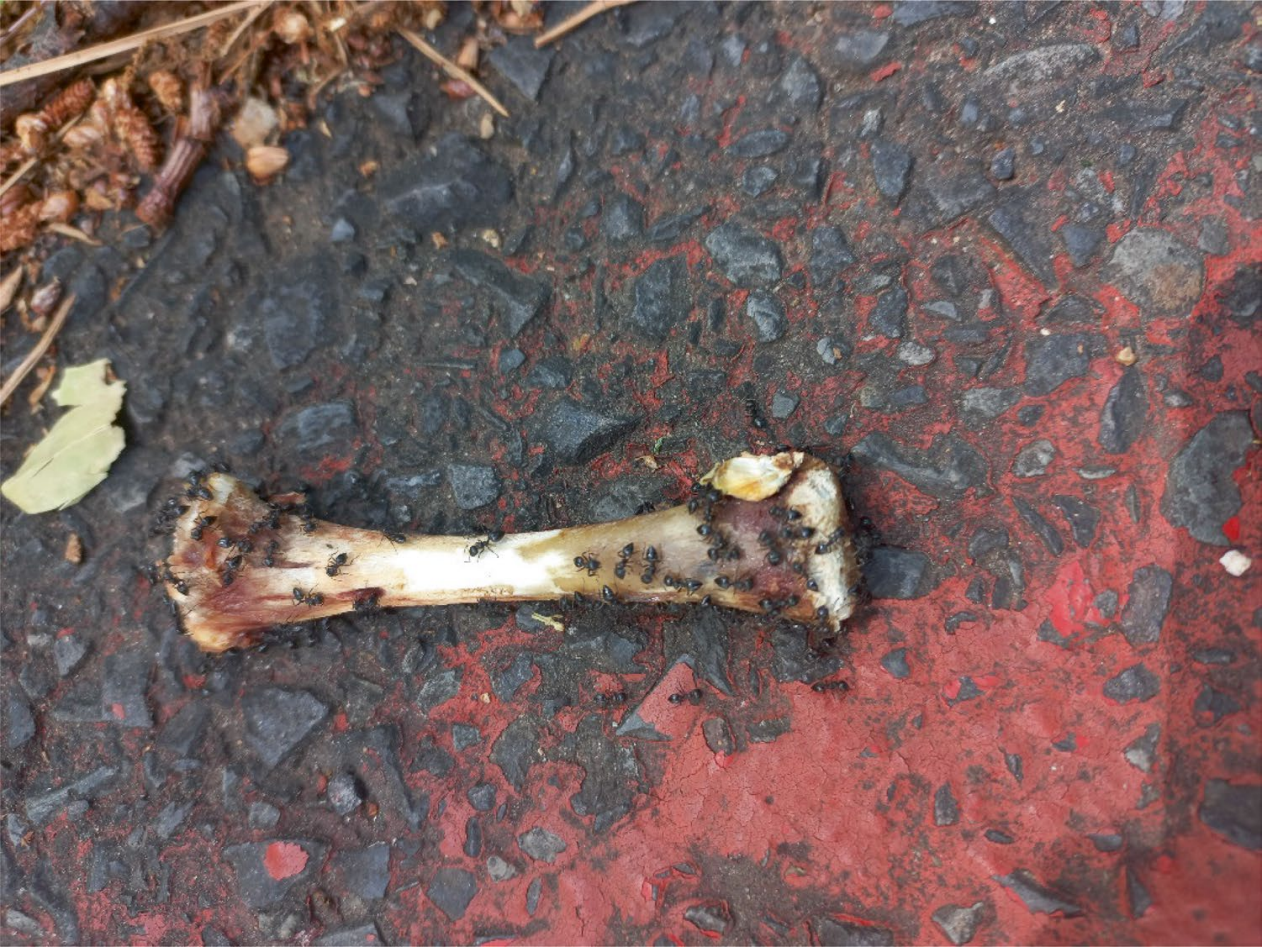
A bone encountered incidentally in the vicinity of the Table Mountain National Park

**Fig. 2:**
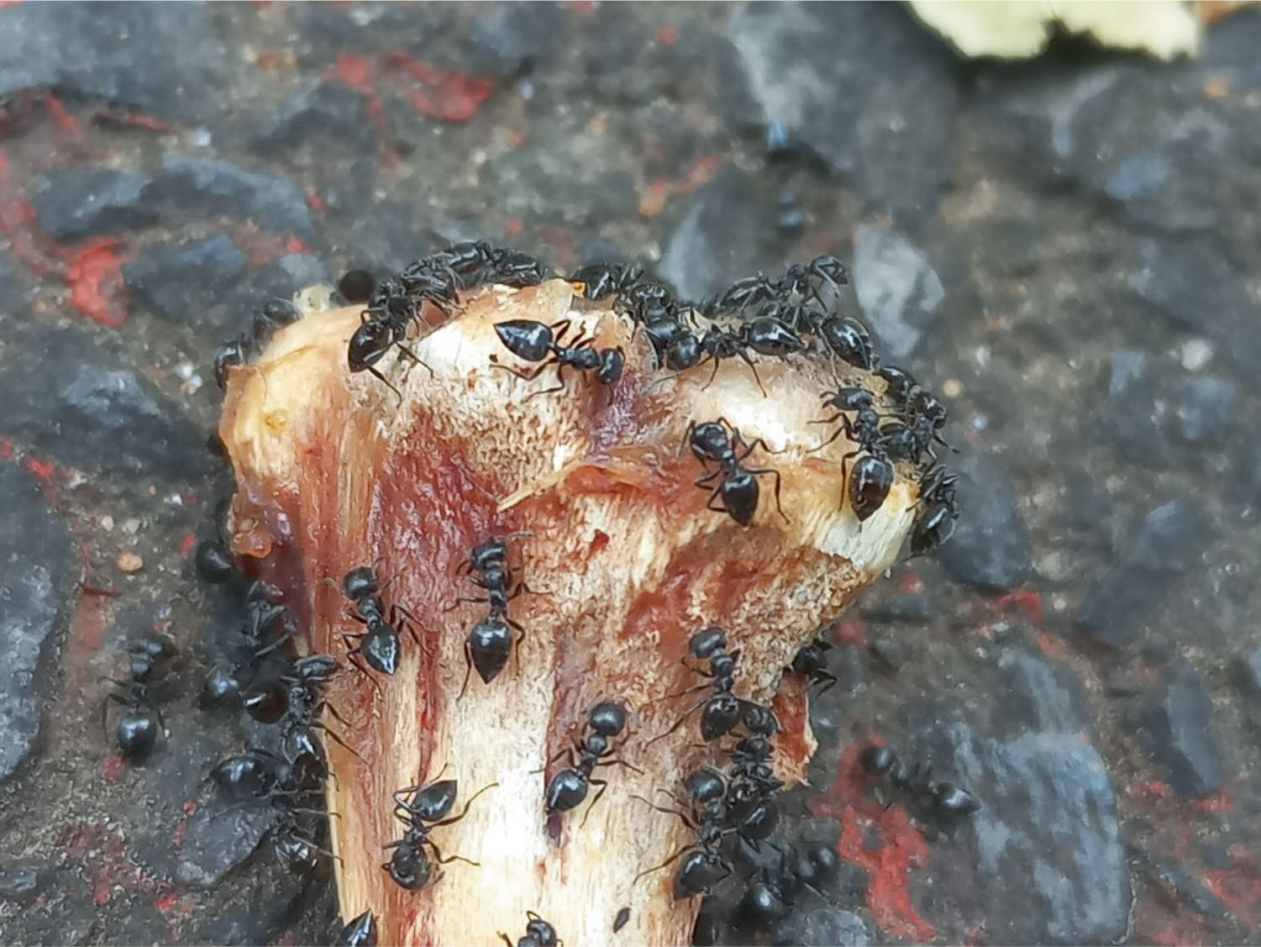
*Crematogaster* cf. *liengmei* individuals aggregating and feeding on the fleshy remnants and bone particles on the anterior part of the bone

**Fig. 3:**
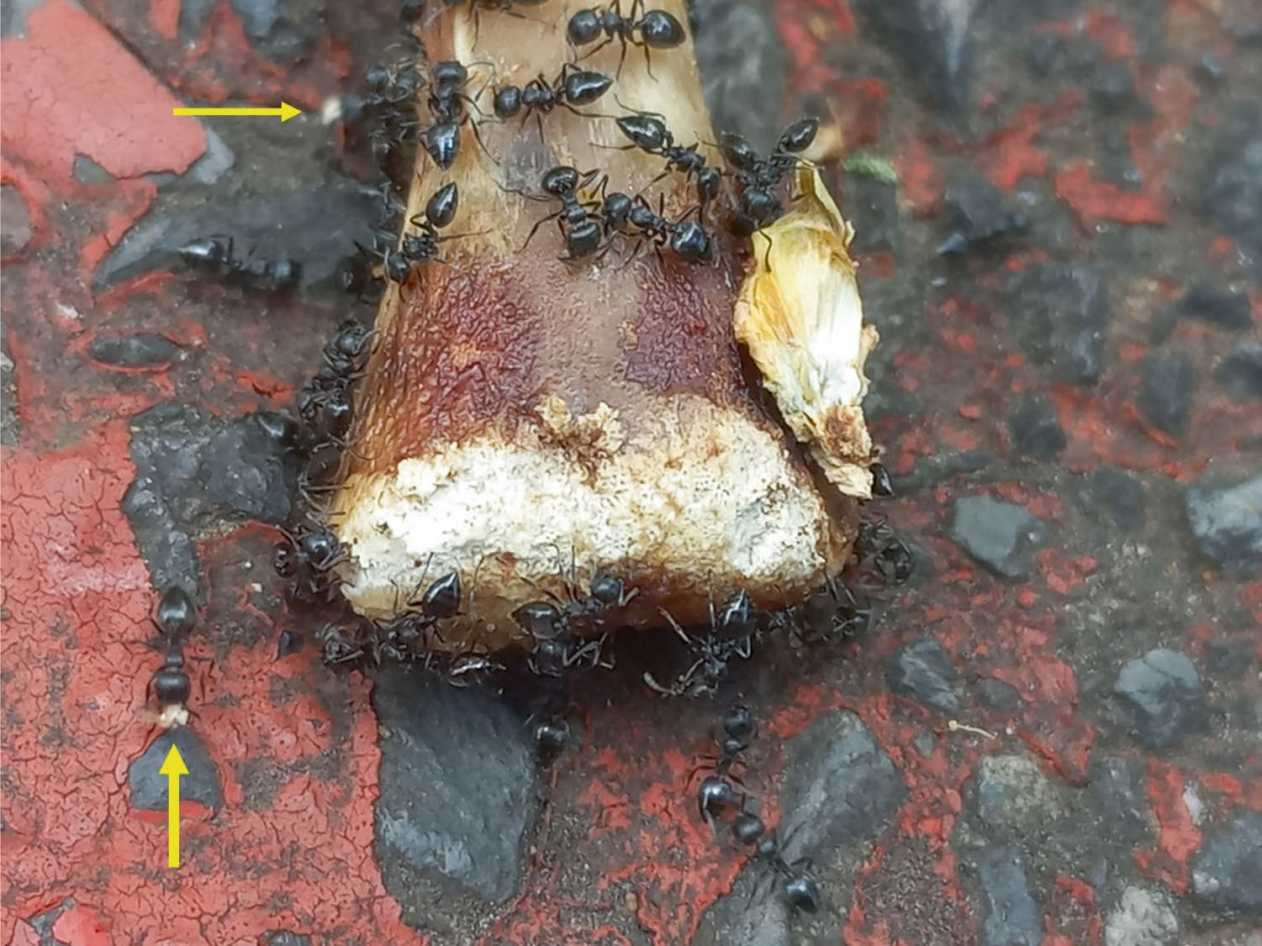
*Crematogaster* cf. *liengmei* individuals aggregating, feeding on, and removing (yellow arrows) fleshy remnants and bone particles on the posterior end of the bone

In November 2022, during routine data collection for an ongoing decomposition and cadaver entomofauna successional study in Table Mountain National Park, a bone was incidentally encountered in the vicinity of the study site (Fig. 1). Upon inspection, it was observed that the bone was colonized by several ants (Fig. 1). A 3-min undisturbed visual observation followed by a short video recording (Online Resource 1) and photography demonstrated active feeding by the ants on the bone. A closer inspection of the bone revealed several striae/furrows at the epiphyseal ends, suspected to originate from the feeding activities of the ants (Figs. 2 and 3). In addition, a few individual ants were observed removing some bone particles and/or soft tissue remnants away from the bone (Fig. 3).

## *Crematogaster *cf. *liengmei* (Hymenoptera: Formicidae) individuals

The ants were identified as *Crematogaster* cf. *liengmei* with the assistance of a local ant specialist using morphological descriptions in Fisher and Bolton [1]. Although identification of the ants to the species level would have been ideal, this is sometimes impossible due to the lack of morphological descriptions and occurrence of morphological variability in insects such as ants [2, 3]. Thus, the abbreviation “cf.” (*confer*, which means “compare to”) indicates the identification is provisional [4, 5].

**Fig. 4:**
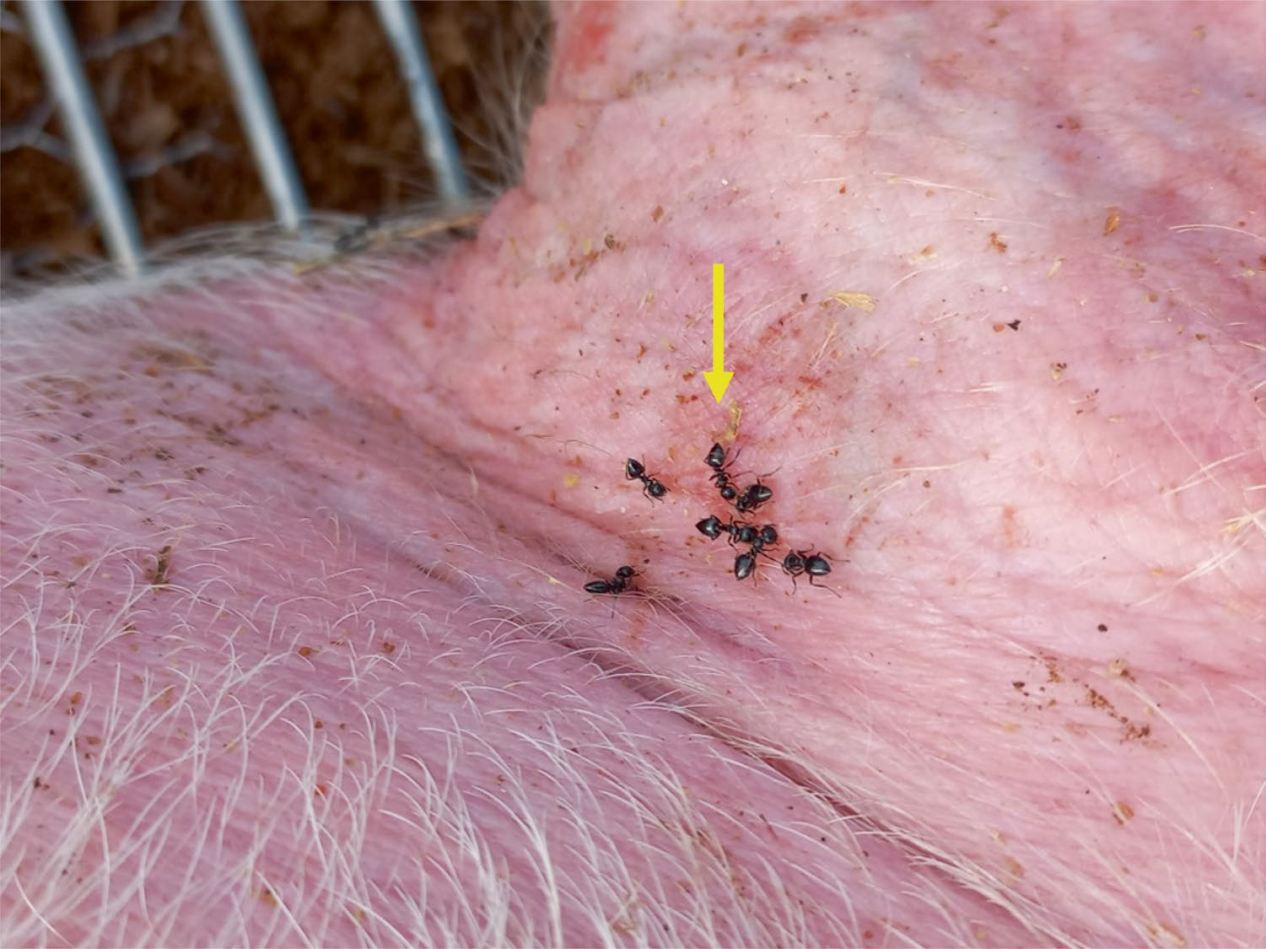
*Crematogaster* cf. *liengmei* individuals aggregating and feeding on the flesh on the external part of the adult pig’s right ear (yellow arrow) as early as day 1 after cadaver deployment

**Fig. 5:**
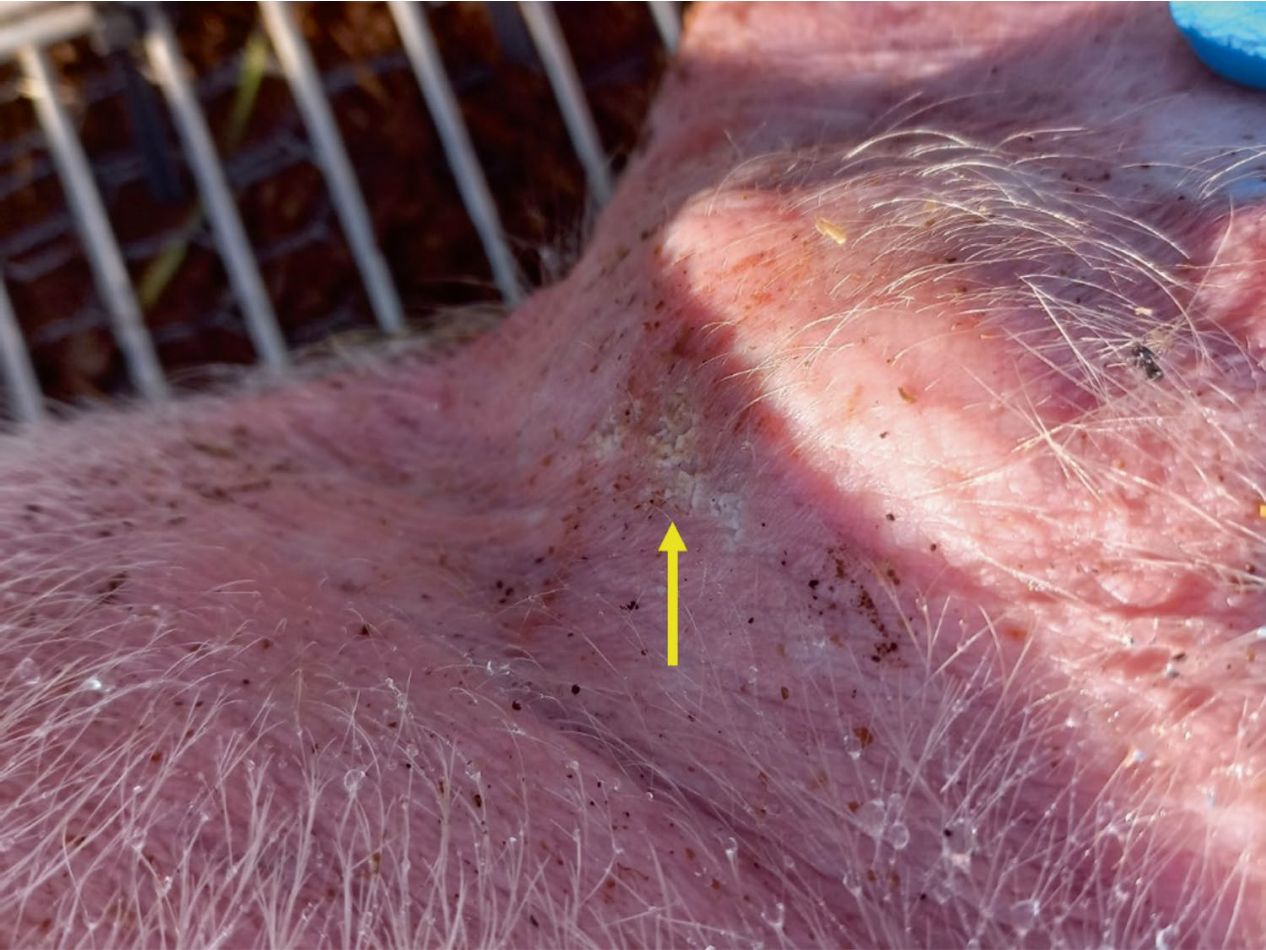
Bitemarks (yellow arrow) inflicted by *Crematogaster* cf. *liengmei* on the external part of the adult pig’s right ear on day 2

The necrophagous behavior of this ant has previously been reported on neonate pig cadavers at the same study site [6]. The morphology of the striae/furrows observed on the bone bears some similarity to the bitemarks seen on the external part of the right ear (Figs. 4 and 5) of an adult pig cadaver, a day after colonization by *Crematogaster* cf. *liengmei*. Due to this similarity, we concluded that the striae/furrows on the bone were created by this same ant species.

## Discussion

The activities of vertebrate scavengers (e.g., vulture, mongoose, raccoon, domestic dog, feral cat, genet, jackal, porcupine, civet, warthog, and rat amongst many others) on vertebrate remains have been extensively investigated and documented in various parts of the world [7–19]. Scavenging activities have the potential to cause significant modification to bodies exposed in the environment, including artefactual disarticulation, and scattering of remains. Such activity alters the rate of decomposition and induces bone modification that may be misconstrued as human-induced ante/peri-mortem trauma.

Invertebrate insect scavengers such as ants have garnered far less attention. Yet their activities on and around vertebrate remains have forensic implications [6, 20, 21]. Globally, several species of ants have been reported to prey on the immature (eggs, larvae, and pupae) and adult stages of other forensically important insects [20–22]. Additionally, they alter soft tissues causing artefacts and hemorrhage, creating sites for adult blow fly oviposition [6, 20–24]. Finally, they prevent fly landing and egg laying on decomposing vertebrate remains [6, 20–22]. These necrophagous and predatory behaviors by ants have been suggested to alter the decomposition of vertebrate remains and the estimation of the minimum time since death when using entomological evidence [20, 21]. Specifically, several members of the genus *Crematogaster* have been reported on decomposing human and animal remains [20, 21] and have been observed to create skin artefacts and alter entomofaunal interactions with a cadaver as described above [25–27]. While several reports exist on the necrophagous and predatory behaviors of various ant species, to the best of our knowledge, only one paper exists on the impact of ants on the skeletal remains of vertebrates [3].

Other invertebrates, including members of the insect orders Isoptera (termites), Coleoptera (beetles), Lepidoptera (moths), and Hymenoptera (wasps and bees), have been documented to feed on and modify the skeletal remains of vertebrates for nutritional purposes and/or the creation of their larval and pupal chambers [28–35]. However, previous reports on the modification of skeletal remains by ants have been anecdotal and speculative [29, 31]. Go [3] is the first author to provide empirical evidence on the contribution of ants as taphonomic bio-agents on vertebrate skeletal remains. During analysis of the skeletal remains of an individual recovered from the Manila North Cemetery in the Philippines, several individuals of *Nylanderia* species (Hymenoptera: Formicidae) were observed nesting in the skeletal remains. Furthermore, post-mortem skeletal alteration in the form of tiny holes, scalloped edges, and shallow striae on several parts of the skeleton (i.e., ankles, tibia, fibula) were attributed to the activities (e.g., gnawing) of the ant species.

The observed striae/furrows on the bone in this study are superficially similar in morphology to the bitemarks/furrows inflicted by some vertebrate [see e.g., 16, 36, 37], and invertebrate (i.e., tenebrionid beetles) [31] scavengers on skeletal remains. These striae/furrows can potentially be misinterpreted as vertebrate scavenging, human-inflicted ante/peri-mortem trauma, or physicochemical weathering. Our incidental observation on the activities of *Crematogaster* cf. *liengmei* provides further evidence on the impact of ants on skeletal remains.

Limited information is available on the biology, ecology, and foraging behavior of *Crematogaster* cf. *liengmei* [6]. Generally, members of the genus *Crematogaster* are known to be tree- and ground-dwelling ants with generalized and omnivorous feeding habits [2, 6, 25]. Their generalized feeding habits may explain why they are able to colonize and feed on the soft tissues of the cadaver and cadaver-associated entomofauna [6]. In addition, they often establish multiple nesting sites in terrestrial environments [2, 6, 25], the proximity of which dictates their ability to immediately colonize vertebrate remains. Above all, the exploitation of vertebrate remains as sources of nutrients by ants, including *Crematogaster* species may be linked to the size and nutritional status of the nests, age and previous foraging experience of the nest inhabitants, and quality of food [6, 25, 38–40].

Congruent with the findings of our previous study [6], we attribute the occurrence of several minute striae/furrows on the epiphyseal ends of the bone to the release of formic acid and other glandular chemicals secreted during the feeding activity of ants. As previously suggested, forensic pathologists, anthropologists, paleobiologists, crime scene investigators, and archaeologists should take cognizance of the presence of ants around skeletal remains of buried and surface decomposing human/animal cadavers. The feeding activity of ants can create striae, furrows, and/or edge gnawings that can mimic vertebrate and invertebrate (e.g., termites and beetles) scavenging, or human-inflicted ante/peri-mortem trauma. It is worth noting in this paper that the morphological state and integrity of the bone prior to its incidental discovery was unknown. Also, the striae/furrows on the bone were observed macroscopically without magnification or microscopic analysis. For these reasons, the possibility of interference by other vertebrate scavengers (e.g., Cape grey mongoose) as documented in Spies et al. [11] and other invertebrate scavengers (e.g., beetles) on the bone prior to our observations cannot be entirely ruled out. Consequently, future field and laboratory-based studies incorporating macroscopic and microscopic analyses will be conducted to provide additional information about the bone modification performed by ants and other invertebrates. This information will be useful in forensic, anthropological, and archaeological investigations.

## Supplementary Information

Below is the link to the electronic supplementary material.

Supplementary file1 (DOCX 13 KB)

## Data Availability

All data generated or analyzed in relation to this study are included in this published article and its supplementary information file.
