# Supplementary material for: Incidental observation of bone modification by Crematogaster cf. liengmei (Hymenoptera: Formicidae) in Cape Town, South Africa
Source: Forensic Sci Med Pathol. 2023 Sep 13;20(3):1121–6. doi: 10.1007/s12024-023-00714-2 (PMC11525404; doi:10.1007/s12024-023-00714-2)
Supplement: Supplementary file 1 — Supplementary file1 (DOCX 13 KB) [file 12024_2023_714_MOESM1_ESM.docx]

**Incidental observation of bone modification by *Crematogaster* cf. *liengmei* (Hymenoptera: Formicidae) in Cape Town, South Africa**

**Supplementary Information File Access Link**: <https://drive.google.com/file/d/1dWtmXgkCibfM8URZdumed971Fvbw1d7k/view?usp=share_link>

**Supplementary Information Caption**

**Online Resource 1** *Crematogaster* cf. *liengmei* individuals actively feeding on and removing bone particles and soft tissue remnants on the bone
